# Correction to: Breaking the barriers of data scarcity in drug–target affinity prediction

**DOI:** 10.1093/bib/bbad477

**Published:** 2023-12-06

This is a correction to: Qizhi Pei, Lijun Wu, Jinhua Zhu, Yingce Xia, Shufang Xie, Tao Qin, Haiguang Liu, Tie-Yan Liu, Rui Yan, Breaking the barriers of data scarcity in drug-target affinity prediction, *Briefings in Bioinformatics*, Volume 24, Issue 6, November 2023, bbad386, https://doi.org/10.1093/bib/bbad386

In the originally published version of this manuscript, there were discrepancies in the affiliations of the authors. The correct affiliations are as follows:

Qizhi Pei: Gaoling School of Artificial Intelligence, Renmin University of China, No.59, Zhong Guan Cun Avenue, Haidian District, 100872, Beijing, China

and

Engineering Research Center of Next-Generation Intelligent Search and Recommendation, Ministry of Education

Tao Qin: Microsoft Research AI4Science, No.5, Dan Ling Street, Haidian District, 100080, Beijing, China

Rui Yan: Gaoling School of Artificial Intelligence, Renmin University of China, No.59, Zhong Guan Cun Avenue, Haidian District, 100872, Beijing, China

and

Beijing Key Laboratory of Big Data Management and Analysis Methods.

This error has been corrected online.

